# A computational theory of short-term synaptic plasticity: synapses learn to tell time

**DOI:** 10.21203/rs.3.rs-9916271/v1

**Published:** 2026-06-19

**Authors:** Saray Soldado-Magraner, Jamie McDowell, Shanglin Zhou, Dean V. Buonomano

**Affiliations:** 1 Departments of Neurobiology and Psychology, University of California, Los Angeles, CA, USA.; 2 Institute for Translational Brain Research, Fudan University, Shanghai, China

## Abstract

In computational neuroscience and deep learning models, synapses are viewed as simple computational elements with a single learnable parameter: synaptic strength. Here we propose that synapses are more sophisticated elements with multiple learnable parameters; allowing synapses to tune their short-term synaptic dynamics and optimize the processing of temporal information. We confirm a prediction of this hypothesis by showing that in mouse and human neocortex, synaptic dynamics is not preferentially shaped by the identity of the pre- or postsynaptic neuron. Using computational approaches, we demonstrate that learned synaptic dynamics allows for interval-selectivity and counting at the synaptic level, and significantly enhances the performance of feedforward networks on complex spatiotemporal tasks. Our results provide a framework to understand the mismatch between the computational simplicity of synapses in models and their biochemical complexity, as well as address a fundamental gap between how the brain and artificial neural networks process temporal information.

## INTRODUCTION

One of the most fundamental principles in neuroscience is that learning relies on long-term changes in synaptic strength.^1–3^ Indeed, this principle has anchored much of the field of machine learning, in which artificial neural networks are trained primarily by tuning the weights of connections.^4–6^ Within this framework, synapses are very simple computational elements as they have only one tunable parameter: the weight. It is well-established, however, that synapses are highly complex biochemical compartments composed of hundreds of different proteins.^7–10^ Moreover, synapses do not actually have a fixed weight on the time scale of a second. Rather, they exhibit robust synaptic dynamics in the form of short-term synaptic plasticity (**STP**), which governs how synaptic strength changes over the course of consecutive presynaptic spikes. Here, we propose that synapses are more sophisticated computational elements than previously recognized, as they may also be able to tune their short-term dynamics in an experience-dependent manner in order to optimize the processing of temporal information.

Short-term synaptic plasticity (**STP**)^11,12^ refers to a form of use-dependent plasticity where a train of presynaptic action potentials can produce progressively smaller (depression) or larger (facilitation) postsynaptic potentials (**PSPs**).^13–18^ While the computational function of STP continues to be debated,^18–21^ experimental and theoretical work indicate that STP contributes to temporal processing on the scale of tens of milliseconds to a few seconds.^17,22–28^ Indeed, these studies propose that it is not coincidental that animal vocalizations, and human speech and music perception are rich in temporal information on the same time scale as STP.^22,24,29,30^

The presence of STP at virtually all neocortical synapses and its potential contribution to temporal processing raises the possibility that some of the biochemical complexity of synapses may be dedicated to tuning the temporal profile of STP. Support for this view comes from studies demonstrating that some presynaptic proteins can alter short-term synaptic dynamics independent of baseline synaptic strength^31,32^—e.g., altering whether a synapse exhibits depression or facilitation while the amplitude of the first PSP remains the same. Here, we propose that rather than merely the first PSP becoming weak or strong via conventional forms of associative and homeostatic plasticity, synapses may also learn to exhibit specific temporal profiles of depression or facilitation to optimize the processing of temporal information. We refer to this hypothesis as Learned Synaptic Dynamics hypothesis (**LSD**). This form of plasticity would help address the apparent mismatch between synapses having a single tunable parameter and the remarkable biochemical complexity of cortical presynaptic terminals—many of which have been associated with cognitive disorders.

The vast majority of neurocomputational studies and machine learning models that use artificial neural networks do not incorporate STP. Even fewer have focused on the possibility that the temporal profile of synaptic dynamics itself could be learned in an experience-dependent fashion. A few models, however, have proposed that presynaptic parameters could be tuned independently from the conventional postsynaptic synaptic weight (see Discussion).^33–37^. Indeed, experimental studies in neocortical synapses have shown that standard forms of associative synaptic plasticity can result in parallel changes in pre- and postsynaptic terminals^38–46^. Under the LSD hypothesis postsynaptic changes govern “baseline” synaptic strength which reflects the static gain of a synapse, while presynaptic changes govern short-term synaptic plasticity, that is in place to specifically optimize the processing of temporal information on the subsecond scale.

## RESULTS

### Synaptic dynamics is shaped by both pre- and postsynaptic neurons

What determines the temporal profile of short-term synaptic plasticity at a given neocortical synapse? Is the temporal profile associated with the identity of the presynaptic neuron and/or of the postsynaptic neuron? Synaptic dynamics is primarily governed by biochemical processes at the presynaptic terminal.^13,14,16,47,48^ But there is a known interaction between long-term associative synaptic plasticity and STP. In the neocortex, but not CA1, long-term potentiation typically enhances short-term depression.^38–43^ A finding consistent with the canonical view that high probability of release synapses favor short-term depression (see Discussion). Some studies, however, are inconsistent with this view; e.g., high probability of release synapses can exhibit facilitation^49,50^ and baseline synaptic strength can be uncorrelated with STP.^44–46^

**Figure 1.**
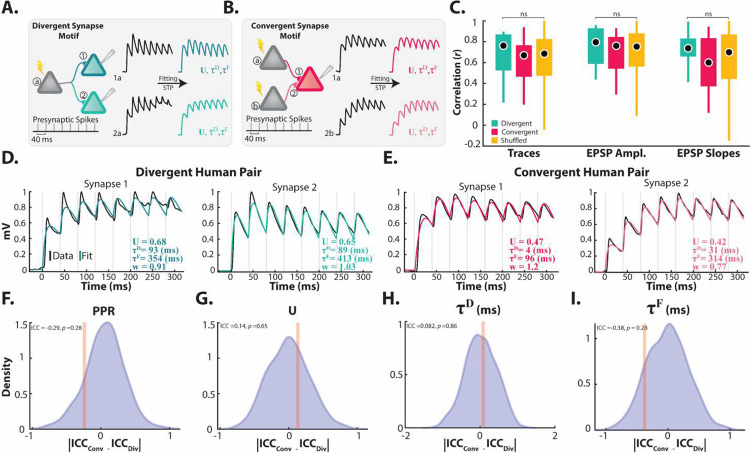
Synaptic dynamics is shaped by both pre- and postsynaptic neuron identity in convergent and divergent triplet motifs. **A-B)** Schematic of divergent (A) and convergent (B) triplet motifs with an example pair of voltage traces and the fitting of the STP parameters *U*, *τ*^*D*^, and *τ*^*F*^. **C)** Pairwise correlation coefficients for voltage traces, amplitudes, and slopes in divergent, convergent sister pairs over the eight evoked EPSPs, and of the pooled shuffled synapse pairs. Boxplots show raw correlation coefficients (*r*). Statistical comparisons were performed on raw correlation values using Wilcoxon rank-sum tests; no significant differences were detected for any feature (*p* > 0.05 for all comparisons). **D-E)** Example voltage-traces (black) and STP fits for a divergent (teal) and convergent (magenta) triplet motif synapse pairs. **F-I)** There was no difference in the intraclass correlation between convergent and divergent synapse pairs for PPR (*p* = .28) (**F**), *U* (*p* = .65) (**G**), *τ*^*D*^ (*p* = .86) (**H**), and *τ*^*F*^ (*p* = 0.28) (**I**). Within-pair correlation was quantified using the intraclass correlation coefficient (ICC), computed across paired synapses within each motif type. Differences in ICC between groups (ΔICC = ICC_Conv_ - ICC_Div_; red line) were tested using a two-sided permutation test. ICC values did not differ significantly between convergent and divergent groups for any parameter.

One way to address the potential contribution of the pre- and postsynaptic neurons to synaptic dynamics is to compare STP between divergent (same presynaptic neuron) and convergent (same postsynaptic neuron) triplet motifs (Fig. 1A–B). To this end, we analyzed the multi-patch recordings in the mouse and human tissue dataset from the Allen Institute.^51,52^ All comparisons were made within the same class of excitatory neurons—e.g., in the divergent-triplets, both postsynaptic neurons were labeled as the same genetically identified subclass (e.g., *Sim1* or *Rorb*), while in the convergent triplets, both presynaptic neurons shared the same label (note that in the human data, excitatory neurons were identified based on layer, morphology, and electrophysiology).

We first asked whether there was any difference in the correlation of voltage traces evoked by a sequence of eight presynaptic spikes (50 Hz), and found no significant difference between the divergent and convergent pairs (*r* = 0.75 and 0.66, respectively, Fig. 1C, **left**). Furthermore, these values were not significantly different from non-triplet pairs (*r* = 0.71). Similarly, there was no difference in the correlation between the EPSP amplitudes (Fig. 1C, **middle**) or slopes (Fig. 1C, **right**) between the convergent and divergent pairs (or with the shuffled non-triplet pairs).

In order to provide a richer quantification of the temporal profile of STP, we fit the voltage traces to the Tsodyks-Markram STP model^53,54^ (Fig. 1D–E). In this model, the temporal profile of STP is governed by three presynaptic parameters: *U*, which is related to the release probability; *τ*^*D*^, which governs the time constant of recovery from vesicle depletion; and *τ*^*F*^, which governs the decay of synaptic facilitation. We calculated the intraclass correlation (**ICC**) of each of these three parameters as well as the paired-pulse ratio (**PPR**) within the divergent and convergent pairs. For the PPR, there was no significant difference between the ICCs of both groups (ICC_Conv_ = −0.087, ICC_Div_ = −0.37, for divergent and convergent pairs, respectively) (Fig. 1F). Similarly, for the STP parameters, there was no significant difference in the ICC values for *U* (Fig. 1G), *τ*^*D*^ (Fig. 1H), or *τ*^*F*^ (Fig. 1I).

Consistent with some previous reports, there was a correlation between the amplitude of the 1^st^ EPSP (EPSP_1_) and PPR (Suppl. Fig. 1). However, there was no correlation between EPSP_1_ and *U*, *τ*^*D*^, or *τ*^*F*^, although *τ*^*D*^ was trending towards significance (p=0.099).

Together, these results show that pre- and postsynaptic identity did not differentially influence the temporal profile of STP across synapses of the same class. Furthermore, initial EPSP amplitude was not correlated with *U*, *τ*^*D*^, or *τ*^*F*^. Indicating that short-term dynamics is not a simple function of baseline strength, and that higher-order relationships between pre- and postsynaptic neurons likely shape synaptic dynamics.

### LSD can be used to solve the Spatiotemporal XOR problem

Historically, simple computational tasks, such as the XOR problem, have played an important role in understanding the limitations and power of neural network architectures and learning rules.^4,55^ In this vein, we first focused on the spatiotemporal-XOR task—which is distinct from the standard “spatial” and temporal XOR tasks.^56,57^

**Figure 2.**
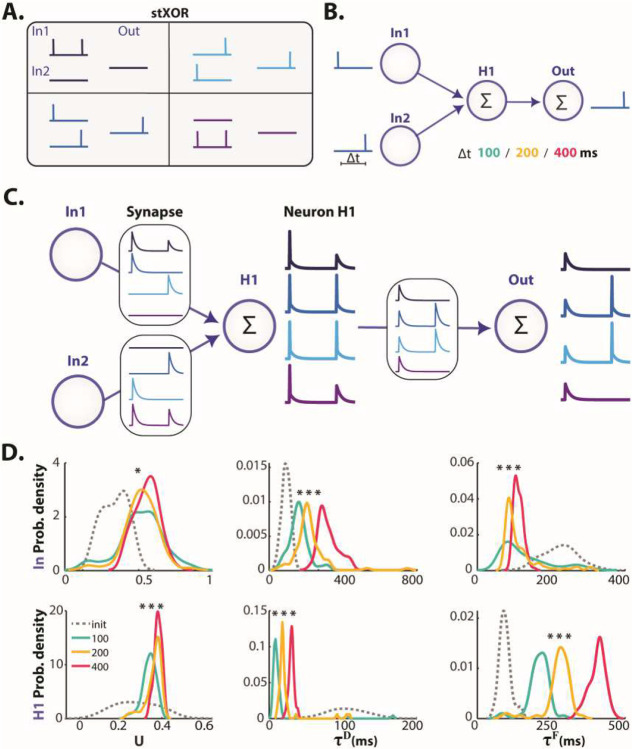
Learned synaptic dynamics enables a minimal network to solve the spatiotemporal XOR task at different intervals. **A)** Schematic of the stXOR task. Two input units (In1 and In2) emit brief pulses separated by a fixed temporal interval (Δt). Output units are trained to produce a spike in response to the In1–In2 and In2–In1 patterns, but not In1–In1 and In2–In2. **B)** Minimal network architecture used to solve the task. Two input neurons project to a single hidden unit (H1), which in turn projects to an output neuron (Out), and all synapses exhibit STP. The delay between the two input spikes (Δt) is set to 100, 200, or 400 ms. **C)** Example dynamics of the trained network illustrating how learned synaptic dynamics implements the solution. Depression at the input synapses prevents the hidden unit firing to repeated pulses (In1-In1; In2-In2). Facilitation at the hidden to output synapse ensures the output unit only responds to the second pulse—allowing the network to selectively activate the output neuron for the target patterns. **D)** Distributions of learned STP parameters after training to each interval. Probability density plots show the distributions of utilization (*U*), depression time constant (*τ*^*D*^), and facilitation time constant (*τ*^*F*^) for the input synapses (In1-2 → H1, top row) and hidden-to-output synapse (H1 → Out, bottom row) after training. Dashed gray curves show the initial parameter distributions, and colored curves show the distributions after training for Δt = 100 ms (green), 200 ms (yellow), and 400 ms (red), with 80, 90 and 100% convergence, respectively. STP parameters systematically adapt to the temporal interval of the task, with longer intervals associated with longer facilitation and depression time constants (In1-2: *U* p=0.03, *τ*^*D*^ p=2.5·10^−41^, *τ*^*F*^ p=0.009, Kruskal–Wallis test, n=50; H1: U p=1.3·10^−10^, *τ*^*D*^ p=6.7·10^−22^, *τ*^*F*^ p=2.7·10^−27^, Kruskal–Wallis test, n=50).

As with the standard XOR task, the spatiotemporal-XOR task has two inputs (A and B) separated by a fixed interval (Fig. 2A). The output neuron should fire once to AB or BA, but not AA or BB. This problem cannot be solved with a conventional integrate-and-fire (**IAF**) network composed of two inputs, a single hidden unit, and an output unit (Fig. 2B). Using this minimal circuit, we implemented STP at each synapse, and each of the three presynaptic STP parameters (*U*, *τ*^*D*^, and *τ*^*F*^) was trained. Thus, together with the weight *w*, each synapse in the circuit possessed four trainable parameters—all of which were trained with surrogate gradients and backpropagation through time.^58,59^ Training converged on a solution in which the two synapses in the first layer exhibited facilitation and the synapse in the second layer exhibited depression (Fig. 2C). Intuitively, the solution is to rely on depression to prevent activation of the hidden unit by the second pulse to the In_1_-In_1_ (AA) or In_2_-In_2_ (BB), and have the output unit fire to the In_1_-In_2_ (AB) or In_2_-In_1_ (BA) as a result of facilitation.

To illustrate the temporal flexibility of LSD, we varied the ISI across one of three intervals: 50, 100, 200 ms, while randomly initializing *U*, *τ*^*D*^, and *τ*^*F*^. Training led to the correct solution, and resulted in differential distributions of the STP parameters for the different intervals (Fig. 2D)—e.g., the *τ*^*D*^ and *τ*^*F*^ parameters were longer for longer ISIs.

### LSD allows single synapses to learn intervals and count

**Figure 3.**
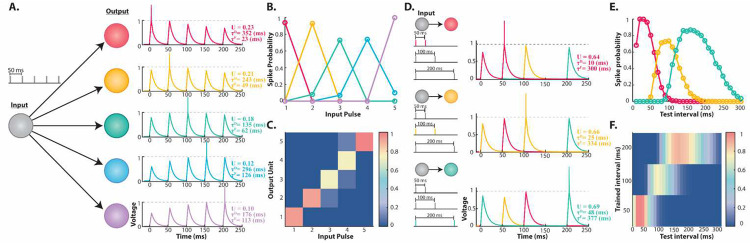
LSD allows single synapses to learn to “count” and underlie interval selectivity. **A)** Schematic of network used for the counting task and voltage responses of output units after training. Each output neuron is trained to selectively respond to a specific input pulse number (magenta = 1, gold = 2, teal = 3, blue = 4, purple = 5). The corresponding short-term plasticity parameters learned by each counting-selective synapse are shown. **B)** Counting tuning curve for output units trained to respond selectively to pulses 1–5 at 20 Hz) The y-axis shows response probability (spikes/trial) across 100 trials with additive noise (σ = 5·10^−3^). **C)** Confusion matrix showing response probability for each output unit (rows) as a function of input pulse number (columns), averaged across 1000 trials (10 seeds × 100 trials per seed). **D)** Schematic of network trained to exhibit interval selectivity. Synapses were trained to elicit a spike in the output units in response to intervals of 50 ms (top, magenta), 100 ms (middle, gold) and 200 ms (bottom, teal). Example voltage response of the trained networks and the corresponding learned STP parameters are shown. **E)** Interval-selectivity tuning curves for synapses trained at 50, 100, or 200 ms. The response probabilities were calculated for each trained synapse across test intervals ranging from 10–500 ms over 100 trials with additive noise (σ = 5·10^−3^). **F)** Confusion matrix showing spike probability as a function of trained interval (rows) and test interval (columns), averaged across 1000 trials (ten seeds × 100 trials per seed).

A computation that requires temporal integration is “counting”: responding selectively to a specific number of consecutive events. Counting neurons have been observed in anuran neurons that respond selectively to the number of auditory pulses at a specific frequency.^60–62^ We next asked if it was possible to tune a postsynaptic unit to respond selectively to a specific number of presynaptic events presented at a fixed inter-spike interval. In this circuit, a single presynaptic unit was connected to five postsynaptic units with STP at each synapse, and stimulated with 5 consecutive spikes (50 ms ISI). Each postsynaptic unit 1 through 5 was trained to respond to spikes 1 through 5, respectively. After training, each unit responded selectively to the target ordinality (Fig. 3A). In the presence of noise, each unit exhibited a tuning curve around its target pulse number, and this tuning was more selective to the extremes, which allowed for a larger solution space (Fig. 3B–C).

Interval discrimination on the subsecond scale is an important computation for many forms of sensory processing, including speech and animal vocalization discrimination, as well as echolocation.^63–66^ Computational and experimental studies have suggested that STP coupled with circuit properties can underlie the formation of interval-selective neurons.^22,24,30,67^ Here, we asked if LSD can account for interval selectivity at the level of a single synapse, in other words, if it is possible to use LSD to train a single synapse to generate a postsynaptic neuron that will respond selectively or preferentially to specific intervals. A single input and output unit was trained to respond selectively to either a 50, 100, or 200 ms interval (Fig. 3D). STP parameters converged to values that generated interval-selective responses as expected, although it should be noted that, as a consequence of inherent constraints of the STP equations used here, there was a relatively small “margin of error” of the final solutions. Even so, in the presence of noise, there was still significant interval tuning toward the target interval (Fig. 3E–F)—a result consistent with a biological examples interval-tuned neurons, which, rather than having a selective response to the preferred interval, exhibits a tuning curve around the preferred interval.^23,68,69^

These results suggest that, at least in principle, counting and interval-selectivity are computations that could be learned at the synaptic level, rather than having to rely on circuit properties, as generally assumed.

### Morse code discrimination in a feed-forward network

**Figure 4.**
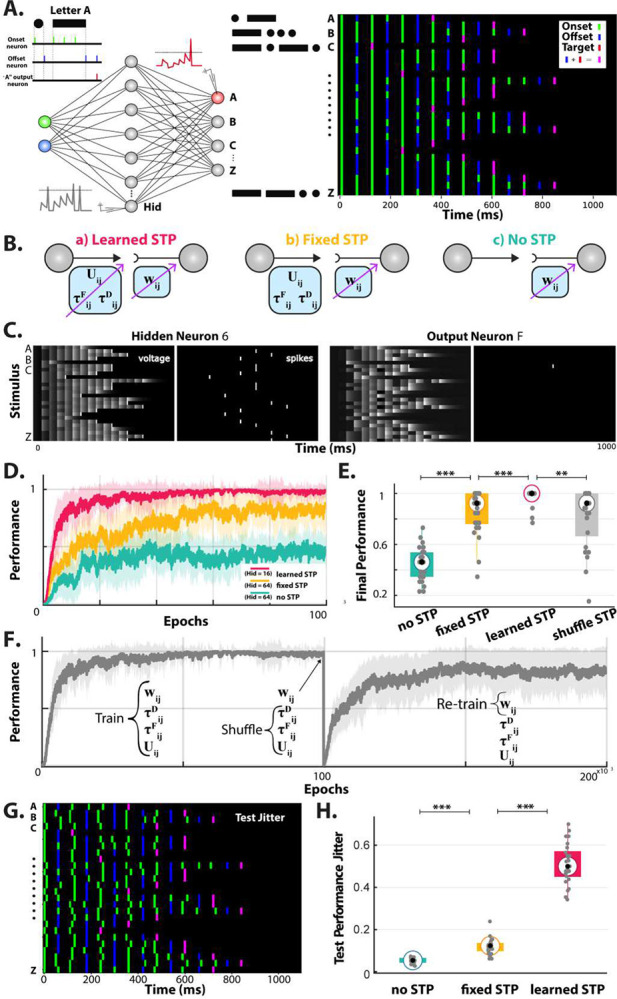
Learned synaptic dynamics enhances Morse code classification and temporal generalization. **A)** Morse code classification task and network architecture. Letters are encoded as temporal sequences of dots (one spike) and dashes (three spikes) delivered through onset and offset input channels. The feedforward spiking network consists of an input layer, a hidden layer, and 26 output neurons corresponding to each letter of the alphabet. The RGB raster plot illustrates the input patterns and the target output neuron corresponding to each letter. The target output time is always at the last offset spike—and thus always in magenta (red + blue). **B)** Synaptic configurations tested across networks. Three architectures were compared: (a) learned short-term plasticity (STP), in which synaptic weights (W) and STP parameters (U, *τ*^*F*^, *τ*^*D*^) were jointly optimized; (b) fixed-STP, where STP parameters were fixed while synaptic weights were trained; and (c) no-STP, where synapses contained only static trainable weights. **C)** Example activity patterns from a trained network with learned-STP. Left panels show membrane voltage and spike activity from a representative hidden neuron across stimulus presentations. Right panel shows output unit “F” in response to all letters. **D)** Training performance across epochs for networks with learned-STP (N_Hid_ = 16), fixed-STP (N_Hid_ = 64), and no-STP (N_Hid_ = 64). Shaded regions represent standard deviation across twenty-five networks. The number of hidden units are adjusted so that they contain the same number of trainable parameters. **E)** Effect of shuffling learned STP parameters. At epoch 100 of training, STP parameters were randomly shuffled across synapses, causing a dramatic drop in performance. Retraining synaptic weights partially restored performance, but it did not reach pre-shuffling performance. **F)** Final classification performance across architectures. Points represent individual network instances and boxes summarize the distribution across networks (interquartile range, 25th–75th percentiles). Learned STP showed higher performance than fixed-STP and shuffled networks (Wilcoxon rank sum, p=1·10^−4^ and p=0.011, n=25), while no-STP was significantly lower than having fixed STP (Wilcoxon rank sum, p=7·10^−8^, n=25) **G)** Example of jittered Morse stimuli used to evaluate temporal generalization. After training, input spike times were temporally jittered to test the robustness across ten trained networks. **H)** Classification performance on jittered stimuli. Networks with learned-STP generalize significantly better to temporally jittered inputs compared to networks with fixed-STP (Wilcoxon rank sum, p=1.4·10^−9^, n=25), while fixed-STP was superior to no-STP networks (p=1.6·10^−8^).

Morse code provides an example of just how sophisticated temporal processing can be in humans. It requires not only the discrimination of the duration of each dot and dash, but the inter-element, inter-character, and inter-word intervals, as well as the number of elements and the overall global temporal structure of the sensory stream. Morse code thus provides a benchmark to quantify the ability of artificial neural networks to process temporal information^70^. Typically, Morse code discrimination would require spatializing time or RNN architectures. We asked if a simple feedforward network with learned synaptic dynamics could classify each letter of the Morse alphabet (Fig. 4A).

Our feed-forward network was composed of two inputs (onset and offset), a single hidden unit layer, and 26 outputs, In addition to the feedforward LSD network with 16 hidden units there were two control networks (Fig. 4B): a no-STP network with 64 hidden units (which matched the number of tunable parameters in the LSD network), and a fixed-STP network with 64 hidden units (which also matched the number of trainable parameters, but had more total parameters than the LSD network). Fig. 4C shows an example of the voltage and spike raster of a hidden unit and output units for all letters. Over the course of training, the performance of twenty-five LSD-networks converged to 100% median accuracy, while the performance of the no-STP network was significantly lower, 46% (Fig. 4D). As expected, the inclusion of fixed STP also significantly improved performance (92%) over the no-STP network by providing a rich temporal reservoir for state-dependent responses,^29,67^ but was significantly below the LSD networks.

Because STP is static in the fixed-STP network, the decreased performance could be due to poor initialization of the STP parameters. To provide a more rigorous control, we first trained the LSD networks until they converged and then shuffled the learned STP parameters across synapses so that STP learning would be disrupted while the distribution of STP parameters remained the same (Fig. 4E). Next, only the *w* parameters were retrained. Performance immediately dropped from 100% to close to chance after shuffling and then converged to approximately 92%, again significantly lower than the original LSD network (Fig. 4F).

To determine whether LSD provides not only superior performance, but also other computational advantages—such as robustness to temporal noise—we tested the above networks on “jittered” Morse code after training (Fig. 4G; see Methods). Here, there was a dramatic difference between the ability of the LSD-network to generalize to jittered stimuli (median 50%) in comparison to the no-STP and fixed-STP controls (5 and 12%, respectively; Fig. 4H)—reflecting the advantage of tuning synaptic dynamics to the temporal structure of the task.

### LSD Enhances Speech Discrimination

**Figure 5.**
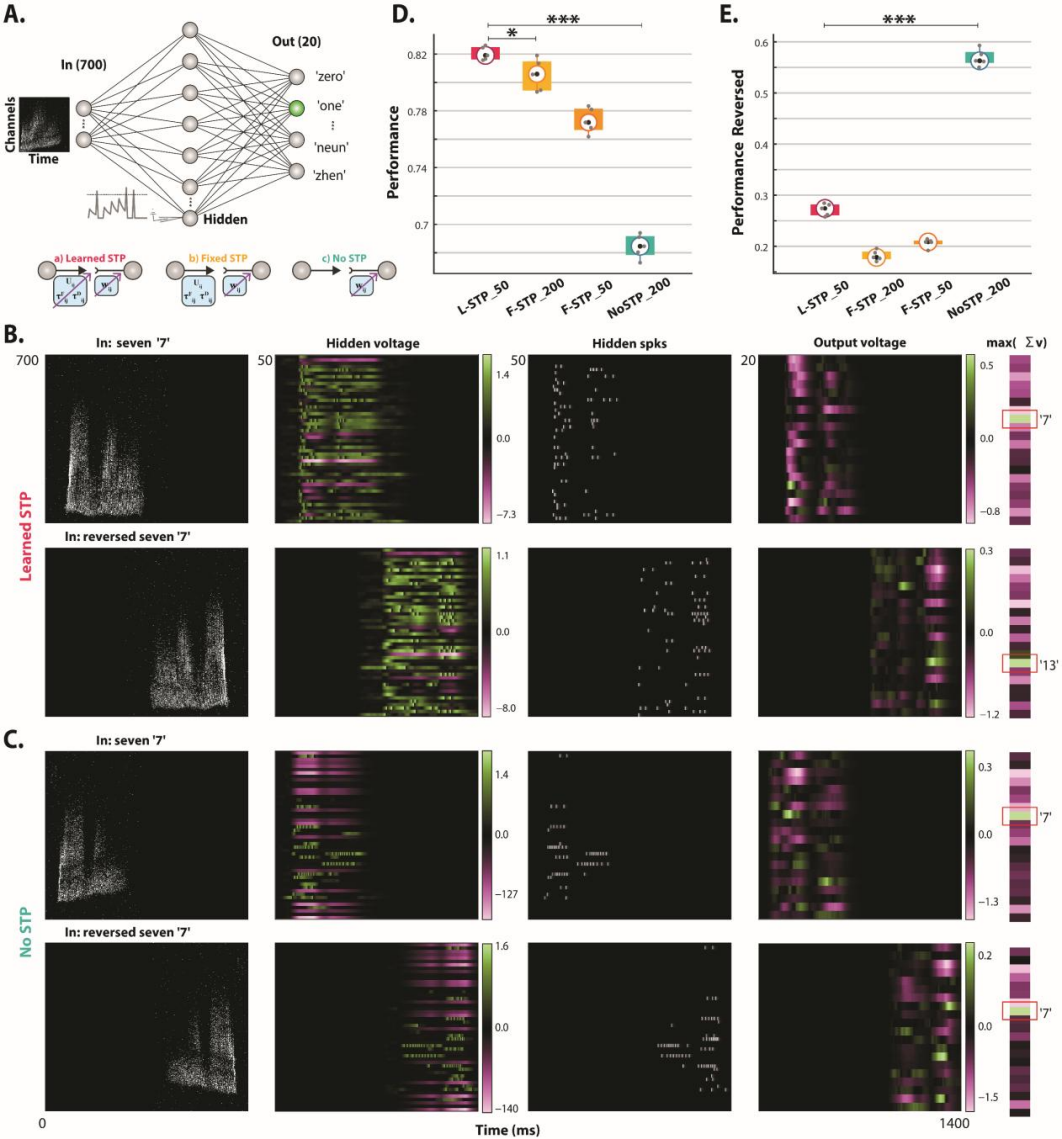
Learned synaptic dynamics improves speech recognition. **A)** Network architecture used for the Heidelberg Spiking Digits (SHD) task. The feedforward spiking network receives spike-based auditory input across 700 channels and classifies each stimulus into one of 20 output classes corresponding to digit identity (0–9) and language (English and German). Synapses are implemented with either learned-STP, fixed-STP, or no-STP. **B)** Example network dynamics for a learned-STP network during presentation of a spoken digit “7” (English). Upper panels from left to right: input spike trains across input channels, hidden layer membrane potentials, resulting hidden layer spike activity, and output neuron voltages. The rightmost panel shows the maximal output response across classes (sum of output voltage across time), correctly identifying the digit. When this same digit is reversed in time (lower panels), the network does not recognize the digit. **C)** Example network dynamics for a network without STP. Panels are organized as in (B). As with the learned-STP case, the network correctly classifies the digit “7”, but in this case, the network also classified the reversed 7 as “7”. **D)** Classification performance across architectures. Networks with learned STP (L-STP, N_Hid_=50) achieve the highest accuracy, followed by networks with fixed STP (F-STP, N_Hid_ = 200) (Wilcoxon rank sum, p=0.032, n=5), while networks without STP (No-STP, N_Hid_=200) show substantially lower performance (Wilcoxon rank sum, p=0.008, n=5). Performance of a fixed STP network with same number of total parameters (50 hidden neurons) is shown for reference (F-STP, N_Hid_=50). **E)** Performance on time-reversed stimuli. When the temporal structure of the speech signals is reversed, networks with STP show a significant drop in accuracy, whereas networks without STP are less affected (No-STP vs L-STP, Wilcoxon rank sum, p=0.008, n=5). This indicates that both trained and fixed STP enables the network to exploit the temporal structure of auditory signals rather than relying primarily on static spatial features.

Speech perception provides a rich example of complex spatiotemporal processing in biological systems, as it requires the integration of information across multiple timescales, including fine temporal structure, spectral features, and sequential organization. To assess whether learned synaptic dynamics can enhance processing of such naturalistic temporal inputs, we evaluated our models on the Heidelberg Spiking Digits (**SHD**) benchmark,^71^ which consists of spike-based auditory representations of spoken digits (0–9) in English and German across multiple speakers. Using a vanilla spiking-RNN composed of 1024 units trained with surrogate-BPTT and data augmentation, performance reaches 83%.^71^ Here, we instead used a purely feedforward spiking LSD-network with a single layer of 50 hidden units (Fig. 5A). Our control networks were composed of no-STP networks with 200 hidden units, and fixed-STP networks with 50 (matching the total number of parameters) and 200 (matching the number of trainable parameters) hidden units. To determine the extent to which the networks were relying on spatial or spatiotemporal information for discrimination, the networks were tested on both the forward and reverse test stimuli.

Figure 5B–C shows an example of an input pattern, hidden activity, and output in an LSD network (Fig. 5B) and a no-STP network (Fig. 5C). In the LSD network, the model correctly classified the forward digit “7”, and correctly failed to recognize the same stimulus when presented in reverse. In contrast, the no-STP network correctly classified the forward digit “7”, but also incorrectly responded to the reversed digit, indicating that it relied primarily on static spatial features of the input rather than its temporal structure.

LSD networks composed of 50 hidden units achieved performance levels similar to the vanilla sRNN (83%)^71^, and performed significantly better than the Fixed-STP_200_ networks. As is common with the SHD and many other benchmarks, the improvement was incremental—with a larger boost in comparison to the Fixed-STP_50_ networks (Fig. 5D). The no-STP networks performed substantially worse. Critically, when evaluated on time-reversed stimuli, both learned-STP and fixed-STP networks showed a marked drop in performance, whereas no-STP networks were significantly less affected (Fig. 5E). This dissociation emphasizes that whether trained or not, STP enables networks to exploit the temporal structure of stimuli. Surprisingly, these results indicate that performance with a feedforward network on a spatiotemporal task was similar to that of an RNN with more parameters.^71^

## DISCUSSION

Convergent research over the past five decades has established long-term synaptic plasticity as the primary mechanism underlying learning and memory.^1–3^ However, it is known that learning can be accompanied by plasticity at other neuronal loci, most notably intrinsic excitability.^72–74^ These findings have profoundly influenced the field of AI; indeed, machine learning models based on artificial neural networks learn by tuning the strength of connections between units and the biases of units—equivalent to synaptic weights and neuronal thresholds, respectively. But other neuronal properties may contribute to the brain’s computational power and to learning. For example, it has been proposed that neurons also exploit the nonlinear electrophysiological characteristics of dendritic arbors and dendritic compartments as computational subunits.^75,76^ Machine learning models have begun to incorporate not only dendritic processing,^77^ but also richer, and plastic neural properties such as membrane time constants,^78,79^ adaptation currents,^80–82^ and synaptic delays^83^.

It remains the case, however, that in the vast majority of neurocomputational and AI models, synapses are represented by a single tunable scalar parameter that captures synaptic strength and does not exhibit any temporal dynamics. This is somewhat surprising given that STP is probably the most experimentally robust form of synaptic plasticity,^13,16^ can be modified by experience,^84–86^ and there are proteins that can modulate synaptic dynamics without affecting baseline synaptic strength.^31,32^

Computational models have proposed that STP contributes to a range of computations, including working memory,^19,87,88^ network stability,^89^ and gain control.^90,91^ Others have proposed that STP contributes to temporal processing,^22,27,30^ and experiments on temporal selective neurons in insects, anurans, fish, and mice support this hypothesis.^23,24,84,92,93^ Even in these cases however, STP has generally been considered a hardwired property that did not adapt to the temporal characteristics of the stimuli being processed. However, a few computational studies explored tuning synaptic dynamics in parallel with the conventional baseline synaptic strength.^35,36^ One recent study demonstrated that training the three presynaptic STP parameters along with the weights of a feedforward network significantly enhanced memory capacity.^37^ Here, we have established that by viewing synapses as computational elements that have more than one tunable parameter, one can significantly enhance the ability of simple circuits to process temporal information. The focus on temporal processing is important because while timing is critical to most forms of sensorimotor processing and cognition,^94,95^ the underlying mechanisms remain to be elucidated.

### Biological Synapses

It is known that the class of both the pre- and postsynaptic neurons shape STP dynamics. For example, Pyr→Pyr connections in the sensory neocortex are generally depressing, while Pyr→SST connections are facilitating.^45,51,96,97^ However, little is known about what governs the high degree of variability in the temporal profile of STP within the same synapse class.^51,98,99^ Baseline synaptic strength between neurons is typically viewed as being determined by associative and homeostatic learning rules, but these rules are generally agnostic to what determines whether a synapse exhibits depression or facilitation, as well as their respective time constants. Because STP is primarily a presynaptic property related to vesicle availability and mobilization, calcium influx, and calcium buffering,^13,14,16,47,48^ it is plausible that synaptic dynamics is shaped by the identity of the presynaptic neuron—e.g., the ratio of specific presynaptic proteins produced in the soma might be homogeneously maintained at all presynaptic terminals. To answer this question, we analyzed convergent and divergent connected triplet motifs (Fig. 1), which revealed that within the same class of synapses, presynaptic identity did not predict STP dynamics any more than postsynaptic identity. This suggests that within a synapse class, STP dynamics of any given synapse is governed by an interaction between the activity patterns of both the pre- and postsynaptic neurons.

In the neocortex, long-term plasticity and can STP interact: long-term potentiation often shifts STP towards depression, while long-term depression shifts STP towards facilitation^38–42^—often resulting in an inverse relationship between baseline synaptic strength and the paired-pulse ratio. This is thought to be because LTP may increase presynaptic release probability in addition to increasing the expression of postsynaptic AMPA receptors.^41,42,100–103^ However, several studies are inconsistent with the standard view that STP is primarily determined by presynaptic release probability: there are presynaptic proteins that can modulate STP in a manner that is uncoupled to initial release probability and baseline synaptic strength^31,32,49^, and baseline synaptic strength can be uncorrelated with STP.^44–46^ Furthermore, this standard view does not address what—if anything—would govern the time constants of recovery from depression and facilitation. Consistent with some previous studies, we show that EPSP_1_ was correlated with PPR (EPSP_2_/EPSP_1_), however, when taking into account the overall temporal dynamics using STP fits, there was no correlation between *U*, *τ*^*D*^, or *τ*^*F*^. These results support the Learned Synaptic Dynamics hypothesis by establishing that overall, STP dynamics is not a simple consequence of the identity of the pre- or postsynaptic neurons, or of baseline EPSP amplitude.

### Learned Synaptic Dynamics

One of the most significant gaps between current ANN implementations in AI and biological neural networks pertains to time: the processing of sequential and temporal information. Transformers are an extreme example in that they are stateless and lack any form of intrinsic dynamics—ordinality is spatially encoded through positional encoding.^104^ But even standard RNNs do not incorporate STP and thus treat synapses as stateless one-parameter elements. A few spike-based models have incorporated STP in feedforward and recurrent architectures and show that it can dramatically enhance discrimination of complex spatiotemporal stimuli.^22,25,67,105^ Indeed, in the early reservoir networks models, STP played an important role in enriching computational power.^22,106^ Here, control studies that relied on fixed-STP further corroborate these findings. This is particularly clear when reverse stimuli are used (Fig. 5)—a standard experimental approach used to determine the true spatiotemporal selectivity of neurons.^107,108^ The Heidelberg benchmark has a significant amount of spatial information: SVMs trained solely on spike counts can reach a performance of 60%.^71^ But the performance of such models drops dramatically when tested on reverse stimuli because output units respond equally well to forward and reverse stimuli—a false positive that humans would never make because reverse stimuli are intelligible.

Learned synaptic dynamics enhanced the ability of a feedforward network to discriminate Morse code letters in comparison to fixed-STP, which already performed well (close to 90%)—but the difference was much more dramatic when generalization to temporally jittered inputs was tested (Fig. 4). Similarly, performance compared to fixed-STP was also better in spoken digit discrimination (Fig. 5). Given the richness of this task, it is particularly surprising that a spiking feedforward network with learned synaptic dynamics reached similar levels of performance as a spiking RNN with more free parameters.^71^ Thus, learned synaptic dynamics provides a potential novel architecture that endows feed-forward networks with some of the same computational abilities as RNNs. Specifically, much as RNNs can store traces of recent input history in their internal dynamics, feedforward networks with STP are also intrinsically able to maintain a recent memory of their input history. Given the substantial computational advantages conferred by LSD, and the diversity of plasticity mechanisms in the brain, it would be surprising if evolution had not already harnessed this mechanism to enhance temporal processing in neocortical circuits.

### Future directions

A critical question relating to the biological plausibility of LSD is that STP is primarily governed by presynaptic mechanisms. Thus, LSD would require a mechanism for retrograde communication from the postsynaptic to presynaptic terminal. This requirement is shared with the already established fact that standard neocortical associative synaptic plasticity has a presynaptic expression component (often revealed by changes in STP).^38–41,44^ While the biochemical underpinning of this retrograde signal remains unclear, several candidate messengers, including endocannabinoids and nitric oxide, have been identified.^41,100,109,110^ Nevertheless, additional research into these potential retrograde signaling mechanisms is required.

A further challenge relates to potential learning rules. As is standard in conventional ANN models where synaptic weights are tuned with stochastic gradient descent, we relied on this method to tune STP parameters. As with the training of weights, this approach is not biologically plausible, leaving unaddressed how LSD could be implemented. However, biologically plausible rules have been put forth regarding how the release probability or the *U* parameter might be learned.^35^ Additionally, it should be noted that with the Tsodyks-Markram implementation of STP, the three presynaptic parameters don’t have rigorous biological equivalents. More biologically detailed models focus on the kinetics of specific presynaptic proteins—such as Synaptotagmin 3 and 7.^111,112^ And it is expected that the regulation of these proteins would have a profound effect on STP. However, much as the biological learning rules underlying tuning of synaptic strength in recurrent circuits remain to be elucidated, the potential mechanisms underlying learned synaptic dynamics must also be the focus of future studies.

## METHODS

### Electrophysiological Analysis of STP Profiles in Triplet Motifs.

To compare the temporal profile of STP of synapses in divergent and convergent triplet motifs, we used the Synaptic Physiology Dataset from the Allen Institute.^51^ For triplet motifs in both mice and humans, we focused on excitatory → excitatory connections that passed the provided “quality check” (qc_pass), and were stimulated using 8 pulses at 50Hz. Note, the standard protocol used by the Allen Institute consisted of 12 pulses, where the first 8 pulses were separated from the last 4 pulses by varying reset intervals. Due to EPSP quality and the limited number of triplet motifs, we focused on the first 8 EPSPs in our analyses and averaged traces across all trials and reset intervals. For convergent motifs, we analyzed 14 mouse pairs (28 synapses) and 7 human pairs (14 synapses). For divergent motifs, we analyzed 9 mouse pairs (18 synapses) and 5 human pairs (10 synapses). Due to noise and unreliable EPSP quality, we excluded 2 pairs from the divergent mouse data and one pair from the convergent mouse data.

### Correlation Statistics

Similarity between the voltage traces, amplitudes, and slopes of triplet motif synapses between convergent, divergent, or shuffled non-sister pairs was quantified as the Pearson correlation coefficient (r) computed across stimulus pulses. To compare similarity distributions between groups (convergent vs. divergent pairs, and sister vs. shuffled non-sister pairs), correlation coefficients were Fisher z-transformed and compared using two-sided rank-sum tests. Shuffled correlations were collapsed across conditions (convergent and divergent) to generate a distribution of independent pairwise correlations.

### Amplitudes and Slopes

To quantify the amplitude and slope of EPSPs, for each PSP (j), we first estimated the maximal rising-phase slope using a sliding-window linear regression procedure. Within the rising phase (from EPSP onset to peak), first-order polynomial fits were computed across overlapping segments of a fixed width. For each segment, the slope coefficient was extracted, and the maximal positive slope across segments was retained. The amplitude of the first EPSP (EPSP_1_) was defined as the maximum membrane potential (peak), within the response window, subtracted from the baseline. To account for temporal summation, for subsequent EPSPs, an exponential decay of the preceding EPSP (j-1) was fit in order to extract a new “baseline”, and amplitudes were calculated by subtracting the maximum membrane potential from the extrapolated baseline.

### STP Parameters

To quantitatively characterize the temporal profile of STP, we fit the voltage traces using a six-parameter model, using the extended version of the Tsodyks-Markram model of STP (Eq. 1 and 2). Model parameters were $\text{x}=\left[U,\tau^{F},\tau^{D},\tau_{\text{mem}},\tau_{\text{syn}},w\right]$, corresponding to the transmitter availability related to release probability (U); the facilitation time constant $\left(\tau^{\text{F}}\right)$; the time constant of recovery from depression $\left(\tau^{D}\right)$; membrane time constant $\left(\tau_{\text{mem}}\right)$; a synaptic current time constant $\left(\tau_{\text{syn}}\right)$, and a postsynaptic weight parameter (w). Model parameters were estimated by nonlinear least squares minimization using MATLAB’S Isqnonlin. Parameter values were constrained to lie within biologically plausible bounds: U[0.05,0.95], $\tau^{F}\left[0.5,500\right]\;\text{ms}$, $\tau^{D}\left[0.5,500\right]\;\text{ms}$, $\tau_{\text{mem}}[1,100]$, $\tau_{\text{syn}}[1,100]$, and w[0.1,100]. To decrease the dependency of the fits on any specific initial parameters, all fits were run over a 5×5×5×1×1×1 grid of initial STP parameters, with U={0.1,0.25,0.5,0.75,0.9}, $\tau^{\text{F}}$ and $\tau^{\text{D}}=\{10,50,100,200,400\}$, $\tau_{\text{mem}}=\{20\}$, $\tau_{\text{syn}}=\{5\}$, and w={1} and fit values with the highest ${\text{R}}^{2}$ were used in the final analyses.

For all STP fits, we focused primarily on an extended form of the Tsodyks-Markram model.^53,54^

(1) $$\frac{dD}{dt}=\frac{\left(1-U\right)}{\tau^{D}}-D\cdot F\cdot \delta \left(t-t_{\text{spike}}\right)$$

(2) $$\frac{dF}{dt}\;=\frac{\left(U-F\right)}{\tau^{F}}+F\cdot (1-F)\cdot \delta \left(t-t_{\text{spike}}\right)$$

The presynaptic spike train at each synapse is represented by $\delta \left(t-t_{\text{spike}}\right)$, where the Kronecker delta δ(t) equals 0 at all values except when there is a presynaptic spike at time t, when it equals 1. The initial values of D and F are 1 and U, respectively. Presynaptic efficacy is determined by the product of D and F. Total synaptic efficacy was obtained by multiplying the presynaptic efficacy by the postsynaptic weight, w.

### Intraclass Correlation Analysis

To quantify within-group similarity of the STP measures, we computed the intraclass correlation coefficient (**ICC**). For each metric, ICCs were computed separately for convergent and divergent groups, yielding $\rho_{A}$ and $\rho_{B}$, and the observed group difference was defined as $\Delta \rho =\rho_{A}-\rho_{B}$. To test whether ICCs differed between convergent and divergent groups, we used a permutation test on Δρ.

### Leaky Integrate-and-Fire Units and Synapses

To assess the temporal capabilities of STP, we evaluated feed-forward artificial neural networks of spiking units (**SNNs**) with synapses that exhibited STP^35^ across multiple temporal tasks and benchmarks. Neurons were modeled as leaky integrate-and-fire (**LIF**) units. The same neuronal and synaptic model was used across all tasks unless otherwise stated. For each neuron, the membrane potential v(t) evolved according to:

(3), $$\tau_{\text{mem}}\frac{dv_{i}(t)}{dt}=-v_{i}(t)+I_{\text{i}}^{Syn}(t)$$

where $\tau_{\text{mem}}$ denotes the membrane time constant and $I_{\text{i}}^{\text{syn}}(t)$ is the total synaptic input current to neuron i. When the membrane potential crossed a fixed threshold ($\theta_{i}=1$), the neuron emitted a spike and its membrane potential was reset (rst =0)

The total synaptic input current entering the membrane equation was:

(4) $$I_{i}^{\text{Syn}}(\text{t})=\sum_{j}w_{ij}^{\text{eff}}(t)s_{j}(t)+\xi_{i}(t)$$

where $s_{j}(t)$ denotes the presence or absence of a presynaptic spike train from neuron j and $\xi_{\text{i}}(t)$ represents additive membrane Gaussian noise.

The effective synaptic weight at each presynaptic spike was given by:

(5) $$w_{\text{ij}}^{eff}(t)=w_{ij}F_{ij}(t)D_{ij}(t)$$

where $w_{ij}$ denotes the baseline synaptic weight from presynaptic neuron j to postsynaptic neuron i and $F_{ij}$ and $D_{ij}$ are the dynamics of facilitation and depression variables.

### Training with Surrogate Gradients

To enable gradient-based training of the non-differentiability of spike events, we relied on surrogate gradient learning. Networks were trained using gradient-based optimization (Adam or Adamax) with separate learning rates for synaptic weights, STP time constants $\left(\tau^{D},\tau^{F}\right)$, and utilization parameters U. Elevated learning rates were used for STP time constants to facilitate learning of temporal dynamics.

Synaptic weights and STP parameters were initialized from bounded random distributions. During training, parameters were constrained to biologically plausible ranges via hard clipping, preventing pathological solutions and stabilizing optimization across tasks. For selected tasks, additional regularization terms penalized excessive spiking activity, including L1 penalties on total spike counts and L2 penalties on per-neuron spike rates.

Gradients were approximated during backpropagation through time using the normalized negative part of a fast sigmoid surrogate. Specifically, we employed the normalized negative part of a fast sigmoid.^59^ The steepness parameter was set to α=1 for the Morse task and to α=100 for the Counting, Interval, and SHD tasks.

The training errors were the mean squared error, except for the SHD task, which minimized the negative log-likelihood of the correct class computed from log-softmax output logits, consistent with standard multi-class classification practice. Detailed hyperparameters, including layer sizes, time constants, learning rates, and optimizer settings for each task, are reported in Supplementary Tables S1–S5.

### Spatiotemporal-XOR task

Two input channels delivered brief pulses separated by a delay $\Delta t\in \left\{100,200,400\right\}\;\text{ms}$. Each interval was tested 50 times across random noise seeds (see Suppl. Table 1 for initialization of parameters). The output target was defined relative to the second pulse. The network architecture was a minimal feedforward circuit with a single hidden and output unit. Membrane noise was set to zero in this task. For computational efficiency, the synaptic depression D and facilitation F variables were updated only at presynaptic spike times $t_{n}$, yielding the closed-form updates:

(7) $$D_{n+1}=1-\left[1-D_{n}\left(1-F_{n}\right)\right]\text{exp}\left(\frac{-\Delta t}{\tau^{D}}\right)$$

(8) $$F_{n+1}=U+\left[F_{n}+F_{n}\left(1-F_{n}\right)-U\right]\text{exp}\left(\frac{-\Delta t}{\tau^{F}}\right)$$

where n indexes presynaptic spike events, and $\Delta t=t_{n+1}-t_{n}$ denotes the inter-spike interval. Membrane potentials were also integrated analytically across inter-event intervals:

(9) $$v_{i}\left(t_{n}\right)=v_{i}\left(t_{n-1}\right)\text{exp}\left(\frac{-\Delta t}{\tau_{mem}}\right)+I_{i}^{syn}\left(t_{n}\right)$$

### Interval and Counting Tasks.

The interval discrimination task required the network to selectively respond to a target inter-stimulus interval presented within a set of possible intervals. Two input pulses were delivered through a single input channel and separated by a delay $\Delta t\in \left\{50,100,200\right\}\;\text{ms}$. All three intervals were presented in a batch. After training, the learned synaptic parameters were tested for robustness by testing the network across a range of inter-stimulus intervals from 10–500 ms in 10 ms increments. Each interval was tested 100 times across random noise seeds, and response probability was calculated as the proportion of trials in which the output unit produced a spike divided by the total number of trials. This was repeated across 10 seeds, and interval tuning curves and confusion matrices were calculated from the average response probability across seeds for a total of 1000 trials (10 seeds × 100 trials per seed).

The counting task required the network to sequentially track the number of input pulses and spike selectively to a target number of input pulses, 1–5, with a fixed inter-pulse interval of 50 ms. The network was made up of one input unit and five output units, corresponding to each of the possible pulse counts. After training, the learned synaptic parameters were evaluated by presenting input sequences seen during training, at varying levels of noise, and each condition was tested 100 times across random noise seeds. Spike probability was calculated as the proportion of trials in which the corresponding output unit generated a spike within the response window, divided by the total number of trials.

For both the interval and counting tasks, membrane noise was normally distributed with ($\mu =0,\;\cdot \sigma =10^{-4}$). Specifically, membrane voltage and STP state variables ($D_{t},F_{t}$) were updated at each time step using forward Euler discretization of the underlying differential equations, rather than relying on analytical closed-form updates between spikes:

(10) $$D_{t}=D_{t-1}+\frac{\Delta t}{\tau^{D}}\left(1-D_{t-1}\right)-F_{t-1}\cdot D_{t-1}\cdot s_{t}\left(1-F_{t}\right)$$

(11) $$F_{t}=F_{t-1}+\frac{\Delta t}{\tau^{F}}\left(U-F_{t-1}\right)+F_{t-1}\left(1-F_{t-1}\right)\cdot s_{t}$$

Where $F_{t}$ is the facilitation variable, $D_{t}$ is the depression variable and $s_{t}$ is the presynaptic input pulse.

### Morse Code Task

For the Morse code task we used a feedforward network composed of two inputs, 16 hidden units, and 26 outputs. Training samples were presented in batches of size 26, corresponding to all Morse letters. The networks were trained to classify the temporally structured spike sequences corresponding to the letters of the alphabet. The input consisted of two channels (onset and offset). Each letter was encoded as a sequence of dots (one spike) and dashes (three spikes) delivered by the onset channel. Dot duration and inter-element intervals were 60 ms, corresponding to a speed of 20 words per minute. At the offset of each dot/dash there was a spike in the offset channel. An additional offset spike was provided after the final element. This is necessary to provide an “end of letter” signal. These offset assumption are consistent with data demonstrating tone-offset neurons in the auditory brainstem^113,114^

As above, for computational efficiency, and given the sparsity of the input spikes, the synaptic depression D and facilitation F variables were updated only at presynaptic spike times $t_{n}$ (Eq. 7–9). For the shuffle control, STP parameters ($U,\tau^{D},\tau^{F}$) were independently shuffled within each layer by applying a full random permutation over all tensor elements (across both pre- and postsynaptic dimensions), thereby preserving marginal parameter distributions while removing any structured assignment of parameters to individual synapses. For the jitter control condition, input spike times were temporally jittered by one time step after training to evaluate robustness across ten independently trained networks.

### Heidelberg Spoken Digit Benchmark.

The SHD task consisted of classifying the Spiking Heidelberg Digits dataset.^71^ Each digit comprises spike trains across 700 input channels over a duration of up to 1.4 s. The classification objective was to assign each stimulus to one of 20 output classes corresponding to digit identity and language. Input spike times were discretized in 400 ms time bins with a time step of 3.5 ms. The network architecture was a feedforward spiking model [700→50→20] with LSD at both input-to-hidden and hidden-to-output connections. Hidden units operated in a spiking regime, while output units integrated activity over time to form class scores. Hidden units were modeled as leaky integrate-and-fire neurons (eq. 3) with synaptic filtering:

(12) $$\alpha =\text{exp}\left(\frac{-\Delta t}{\tau_{syn}}\right)$$

Where $\tau_{\text{syn}}$ corresponds to the synaptic time constant. Membrane voltage and synaptic state variables were updated at each time step using forward Euler discretization. Output neuron activity was summed over time to obtain a single scalar value per class, which served as the unnormalized output scores (logits). Logits were passed through a log-softmax transformation, and networks were trained by minimizing the negative log-likelihood (**NLL**) of the correct class. Additional regularization terms penalized excessive spiking activity via L1 and L2 spike-count penalties.

## Supplementary Material

1

## Data Availability

All code will be available upon publication at https://github.com/saraysoldado/LearnedSTP.git. The SHD dataset is publicly available under https://zenkelab.org/resources/spiking-heidelbergdatasets-shd/
