# Supplementary material for: A computational theory of short-term synaptic plasticity: synapses learn to tell time
Source: Res Sq. 2026 Jun 19:rs.3.rs-9916271. Preprint. [Version 1] doi: 10.21203/rs.3.rs-9916271/v1 (PMC13308365; doi:10.21203/rs.3.rs-9916271/v1)
Supplement: 1 [file NIHPPRS9916271V1-supplement-1.pdf]

Supplementary Materials

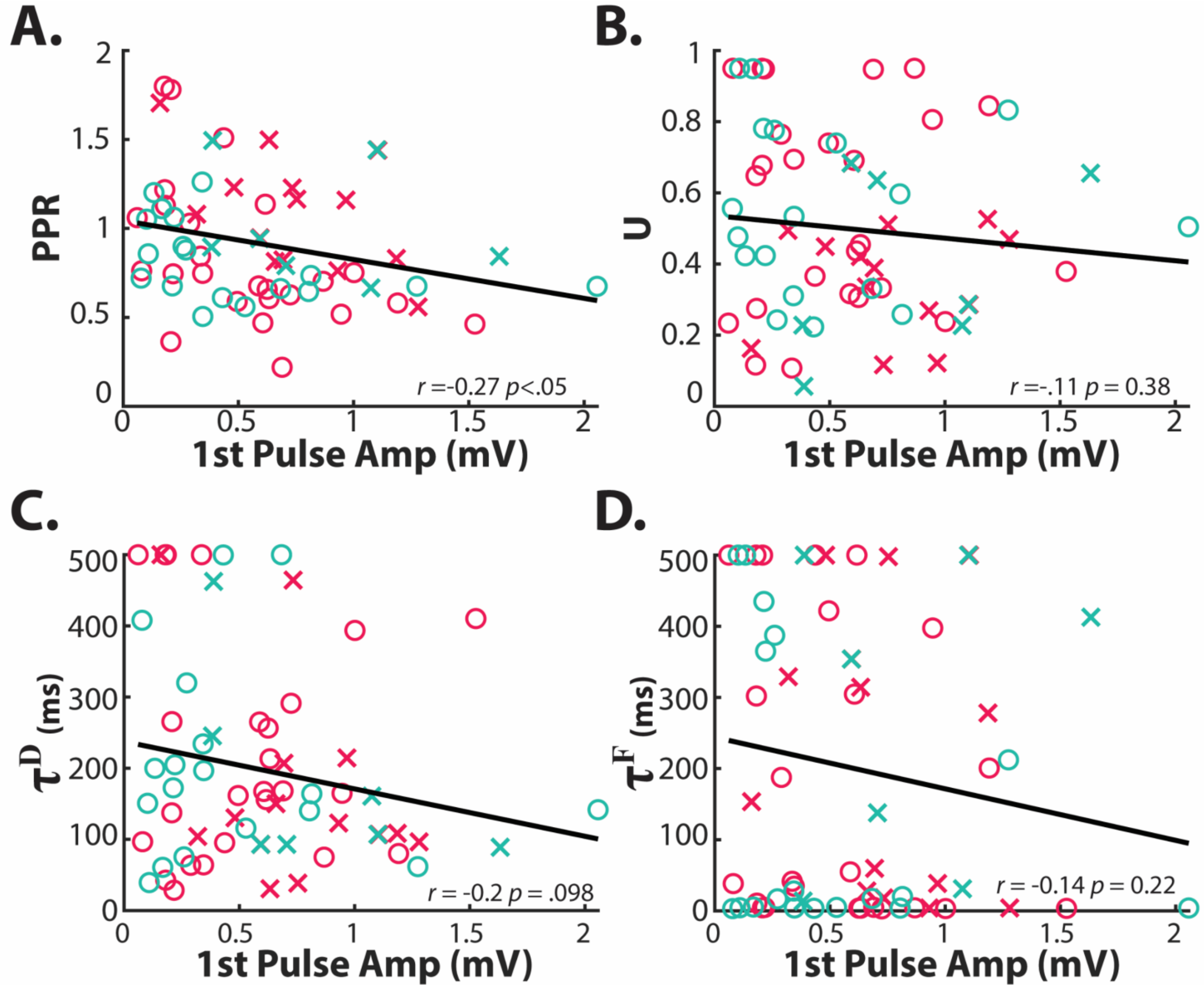

Supplementary Figure S1. Relationship between baseline EPSP amplitude and STP parameters across synapses. **A-D)** Relationship between first-pulse EPSP (**$EPSP_1$**) amplitude and STP metrics. **A.** $EPSP_1$ amplitude is plotted against (**A**) paired-pulse ratio ($PPR=EPSP_2/EPSP_1$); (**B**) Utilization parameter, U; (**C**) depression time constant, τ^D; (**D**) and facilitation time constant τ^F for convergent (magenta) and divergent (teal) synapses. Mouse and human synapses are indicated by circles and x's, respectively. Black lines denote linear fits to pooled data across groups. Pearson's r correlation coefficients were computed for each predictor. A significant negative correlation was observed between $EPSP_1$ amplitude and PPR (Pearson's r = -0.27, p<0.05).

Supplementary Table S1. Spatiotemporal XOR (stXOR) task parameters

Parameter	Value
Input neurons	2
Hidden neurons	1
Output neurons	1
Network architecture	[2 → 1 → 1]
Delay intervals	100, 200, 400 ms
Stimulus onset	10 ms
Stimulus duration	1 ms
Response window	1 ms
Membrane time constant τ_{mem}	10 ms
Utilization U	U(0.05, 0.95)
Depression time constant τ_d	U(50, 500) ms
Facilitation time constant τ_f	U(50, 500) ms
Depression and Facilitation (τ_d, τ_f) Clip	[5, 1000] ms
Utilization U Clip	[0.05, 0.95]
Weights w Clip	[0.01, 100]
Noise σ	0
**Hyperparameter**	**Value**
Loss function	MSE
Optimizer	Adam
Learning rate (w)	1×10^{-3}
Learning rate (τ_d, τ_f)	×100
Batch size	4
Max training trials	50,000

Supplementary Table S2. Interval task parameters

Parameter	Value
Input neurons	1
Output neurons	1
Network architecture	[1 → 1]
Delay intervals	50, 100, 200 ms
Stimulus onset	10 ms
Stimulus duration	1 ms
Response window	1 ms
Membrane time constant τ_{mem}	10 ms
Utilization U	U(0.05, 0.95)
Depression time constant τ_d	U(100, 200) ms
Facilitation time constant τ_f	U(100, 200) ms
Depression and Facilitation (τ_d, τ_f) Clip	[5, 1000] ms
Utilization U Clip	[0.05, 0.95]
Testing Intervals	{10:10:500 ms}
Training noise σ	$1*10^{-4}$
Testing noise σ	[$1*10^{-4}$, $1*10^{-3}$, $5*10^{-3}$, $1*10^{-2}$]
**Hyperparameter**	**Value**
Loss function	RMSE
Optimizer	Adam
Learning rate (w)	5×10^{-3}
Learning rate (τ_d, τ_f)	×100
Batch size	3
Max training trials	20,000

Supplementary Table S3. Counting task parameters

Parameter	Value
Input neurons	1
Output neurons	5
Network architecture	[1 → 5]
Delay intervals	50 ms
Stimulus onset	10 ms
Stimulus duration	1 ms
Response window	1 ms
Membrane time constant τ_{mem}	10 ms
Utilization U	U(0.05, 0.95)
Depression time constant τ_d	U(100, 200) ms
Facilitation time constant τ_f	U(100, 200) ms
Depression and Facilitation (τ_d, τ_f) Clip	[5, 1000] ms
Utilization U Clip	[0.05, 0.95]
Training Noise σ	$1*10^{-4}$
Testing noise σ	[$1*10^{-4}$, $1*10^{-3}$, $5*10^{-3}$, $1*10^{-2}$]
**Hyperparameter**	**Value**
Loss function	RMSE
Optimizer	Adam
Learning rate (w)	1×10^{-3}
Learning rate (U, τ_d, τ_f)	x1
Batch size	1
Max training trials	20,000

Supplementary Table S4. Morse code recognition task parameters

Parameter	Value
Input neurons	2
Hidden neurons	16
Output neurons	26
Network architecture	[2 → 16 → 26]
Time step	10 ms
Total duration	1100 ms
Dot duration	60 ms
Inter-element interval	60 ms
Membrane time constant τ_{mem}	50 ms
Utilization U	U(0.05, 0.95)
Depression time constant τ_d	N(100, 25) ms
Facilitation time constant τ_f	N(100, 25) ms
Depression and Facilitation (τ_d, τ_f) Clip	[5, 1000] ms
Utilization U Clip	[0.05, 0.95]
Noise σ	$1\text{x}10^{-5}$
**Hyperparameter**	**Value**
Loss function	MSE
Optimizer	Adam
Learning rate (w)	1×10^{-3}
Learning rate (τ_d, τ_f)	×500
Learning rate (U)	×0.1
Batch size	26
Max training trials	100000

Supplementary Table S5. Heidelberg Spiking Digits (SHD) task parameters

Parameter	Value
Input neurons	700
Hidden neurons	50
Output neurons	20
Network architecture	[700 → 50 → 20]
Max stimulus duration	1.4 s
Time step	3.5 ms
Time steps	400
Membrane time constant τ_{mem}	35 ms
Synaptic time constant τ_{syn}	17.5 ms
Utilization U	U(0.05, 0.95)
Depression time constant τ_d	U(50, 500) ms
Facilitation time constant τ_f	U(50, 500) ms
Depression and Facilitation (τ_d, τ_f) Clip	[50, 1000] ms
Utilization U Clip	[0.05, 0.95]
Noise σ	0
**Hyperparameter**	**Value**
Loss function + Regularization	NLL (L1 + L2 on spikes)
Optimizer	Adamax
Learning rate (w)	2×10^{-4}
Learning rate (τ_d, τ_f)	×50
Learning rate (U)	×0.1
Batch size	32
Max training trials	100
